# 7/m – Sturz vom Baum: Vorbereitung auf die Facharztprüfung: Fall 78

**DOI:** 10.1007/s00113-021-00986-9

**Published:** 2021-03-25

**Authors:** Annelie-Martina Weinberg, Christoph Stotter

**Affiliations:** 1 Universitätsklinik für Orthopädie und Unfallchirurgie, MUG Graz, Auenbruggerplatz 5, 8034 Graz, Österreich; 2 Abteilung für Orthopädie & Traumatologie, Landesklinikum Baden-Mödling, Sr. M. Restituta-Gasse 12, 2340 Mödling, Österreich

**Keywords:** Humerusfrakturen, Durchblutung, Diagnostische Bildgebung, Frakturosteosynthese, Postoperative Komplikationen

## Prüfungssimulation

### Fallschilderung

Ein 7‑jähriger Junge wird nach einem Sturz von einem Baum ins Krankenhaus eingeliefert. Im Bereich des rechten Ellenbogens gibt der Junge starke Schmerzen an. Eine offene Verletzung besteht nicht. Durchblutung, Motorik und Sensibilität sind präklinisch nicht valide prüfbar. Von den Notfallsanitätern wurde eine Ruhigstellung in Form einer anpassbaren Schiene am rechten Ellenbogen vorgenommen.

## Prüfungsfragen

- Gehen Sie auf die klinische Erstversorgung ein.
- Welche radiologische Diagnostik streben Sie an? Benennen Sie die Verletzung.
- Nennen Sie die Klassifikation der vorliegenden Verletzung, und klassifizieren Sie die Verletzung des vorliegenden Falls.
- Welches Korrekturpotenzial weist die Verletzung im Kindesalter auf?
- Welche Therapieoptionen stehen unter Berücksichtigung des Korrekturpotenzials und der unterschiedlichen Frakturtypen zur Verfügung? Welche wählen Sie im konkreten Fall?
- Welche posttraumatischen Komplikationen können eintreten?

### Antworten

#### Gehen Sie auf die klinische Erstversorgung ein.

- Reevaluation der korrekten Immobilisation mit Einschluss des Handgelenks.
- Anamnese:
  - **Allergien**: v. a. zur Planung medikamentöser und operativer Therapien.
- Prüfung von Durchblutung, Motorik und Sensibilität:
  - **neuro-/motorischer Status**: Sensible Schäden sind häufig initial erschwert differenziert zu untersuchen. Zur Prüfung der Motorik kann der Patient richtungweisend aufgefordert werden, Daumen, Zeige- und Mittelfinger zu strecken, ein „O“ mit Daumen und Zeigefinger zu formen, einen Faustschluss durchzuführen sowie die Hand dorsal zu flektieren. Dies ist bei fast jedem Patienten möglich.
  - **Gefäßstatus**: Eine mangelnde Durchblutung stellt eine Notfallindikation dar. Bei Minderperfusion sind das schnelle Erkennen und die Wiederherstellung der Perfusion die obersten Ziele der Behandlung. Da das dislozierte Gelenk oder der dislozierte Knochen die häufigsten temporären Ursachen sind, führt die Reposition oftmals sofort zur sofortigen Reperfusion.
- Ausschluss und Reevaluation eines sich entwickelnden Kompartmentsyndroms.
- Analgetikagabe (Suppositorien, i.v.- oder nasale/bukkale Applikation).
- Untersuchung auf Zeichen einer offenen Fraktur:
  - Infektionsprophylaxe bei offenen Frakturen [1],
  - Evaluation des Tetanusschutzes.
- Dokumentation der erhobenen Maßnahmen.
- Kindgerechte Betreuung und Erklärung der Maßnahmen.
- Enge Einbindung und Aufklärung der Erziehungsberechtigten.

##### Der Fall.

Der Patient weist zwar, soweit beurteilbar, eine intakte Sensibilität auf, aber die Hand ist in der klinischen Untersuchung im Bereich der A. radialis und der A. ulnaris nicht mehr durchblutet. Es handelt sich um eine geschlossene Fraktur. Weitere Verletzungen liegen nicht vor.

#### Welche radiologische Diagnostik streben Sie an? Benennen Sie die Verletzung.

**Figure 1:**
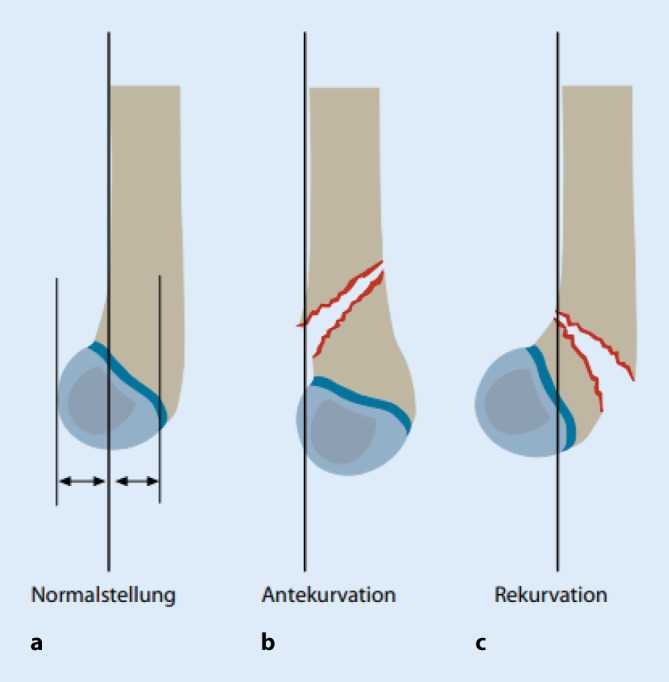

Als Frakturdiagnostik ist zunächst die konventionelle Röntgenbildgebung des Ellenbogens in 2 Ebenen ausreichend. Folgende radiologische Zeichen sind zu beachten:

- vorderes und hinteres Fettpolsterzeichen,
- Rogers-Hilfslinie im seitlichen Röntgenbild (Abb. 1 und 2),
- Rotationssporn: Kalibersprung im seitlichen Bild [2].

**Figure 2:**
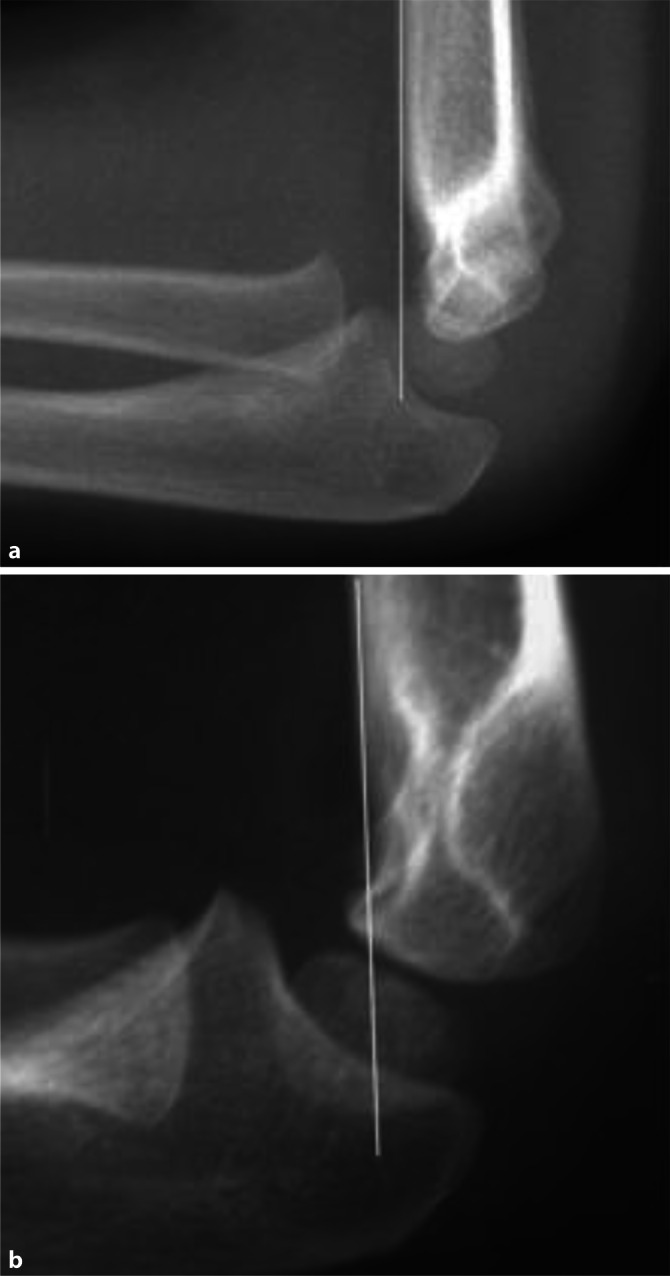

Diagnostische Schwierigkeiten können wie folgt subsumiert werden:

- radiologischer Nachweis nicht- oder minimal dislozierter Frakturen,
- Abgrenzung zur Wachstumsfugenverletzung,
- Erkennen einer Rotationsabweichung (Zeichen der Instabilität),
- Abgrenzung einer Knochenzyste oder Osteolyse.

##### Der Fall.

**Figure 3:**
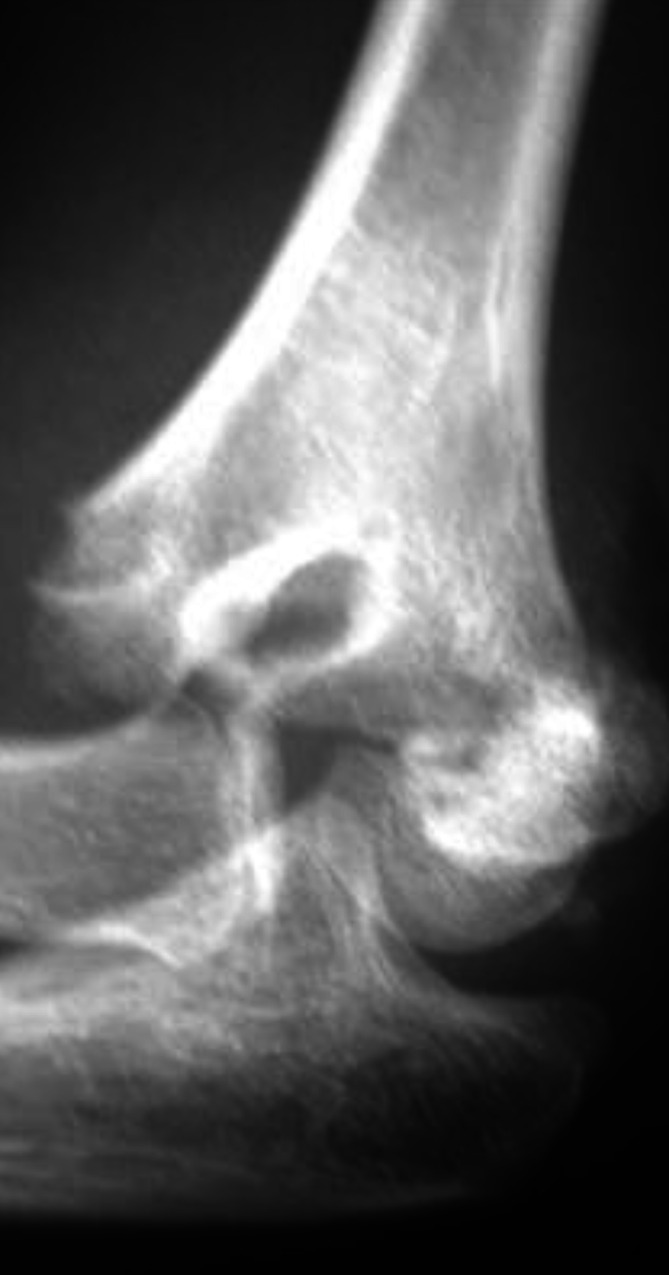

Es erfolgt die sofortige Röntgenbildgebung des Ellenbogengelenks; schmerzbedingt ist lediglich eine Ebene durchführbar (Abb. 3).

**Figure 4:**
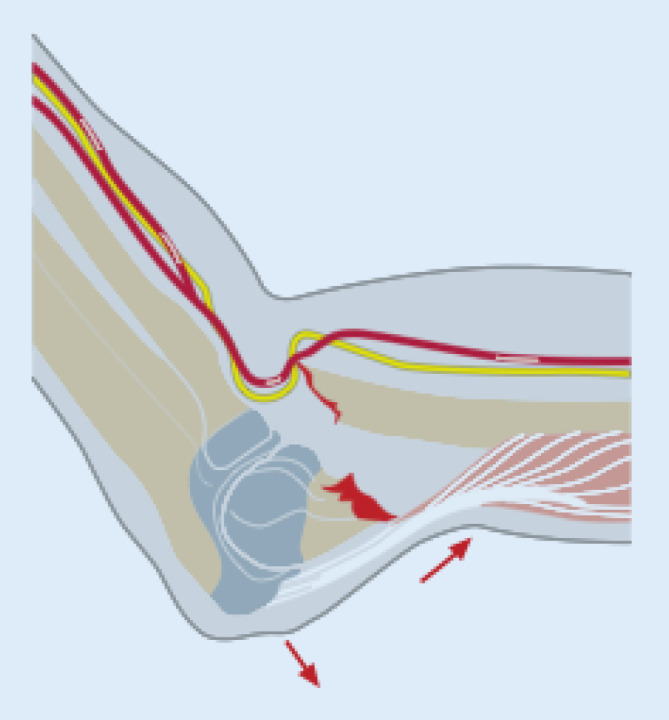

Es zeigt sich eine dislozierte suprakondyläre Humerusfraktur. Bei dem zusätzlichen klinischen Verdacht auf eine Gefäßläsion im Frakturbereich (Abb. 4) wurde die Notfallindikation zur Operation gestellt und der Patient unmittelbar in den OP verbracht.

#### Nennen Sie die Klassifikation der vorliegenden Verletzung, und klassifizieren Sie die Verletzung des vorliegenden Falls.

Man unterscheidet die Extensionsfraktur (98 % der Fälle), die eine Antekurvationsfehlstellung beinhaltet und einen ventralen Sporn aufweisen kann, von den Flexionsfrakturen (2 % der Fälle), die durch eine Rekurvationsfehlstellung mit/ohne dorsalen Sporn gekennzeichnet sind. Zahlreiche Klassifikationen wurden in der Literatur beschrieben.

Die empfohlene und gebräuchliche Klassifikation [1] ist die AO/OTA-Klassifikation für Frakturen der langen Röhrenknochen im Kindesalter, die die Klassifikation nach van Laer übernommen hat und der ein Behandlungsalgorithmus folgt [1, 9, 10].

- Typ I: keine Dislokation,
- Typ II: Dislokation in einer Ebene,
- Typ III: Dislokation in 2 Ebenen,
- Typ IV: Dislokation in 3 Ebenen oder vollständige Dislokation.

International – v. a. im angloamerikanischen Raum – wird häufig die Gartland-Klassifikation angewendet [3, 4].

##### Der Fall.

Aufgrund der vollständigen Dislokation wird eine Fraktur vom Typ IV nach van Laer klassifiziert.

#### Welches Korrekturpotenzial weist die Verletzung im Kindesalter auf?

Bei der Behandlung der suprakondylären Oberarmfraktur kann die Möglichkeit der **Spontankorrektur** in die Therapie einbezogen werden, wobei diese nur in der Sagittalebene zu erwarten ist. Sie ist bis zum 7 bis 8. Lebensjahr begrenzt, da der Ellenbogen schon früh keine Wachstumspotenz mehr aufweist. Dies bedeutet, dass Achsfehlstellungen nach **Extensionsfrakturen** in der seitlichen Ebene ab dem 6. Lebensjahr nicht mehr belassen werden dürfen (Rogers-Hilfslinie, Abb. 2). **Flexionsfrakturen** sind instabil und werden daher meist nicht dem spontanen Remodeling überlassen.

Fehlstellungen in der Frontalebene (Cubitus varus und valgus) korrigieren sich **nicht** und sind ebenso wie Rotationsfehlstellungen (zu erkennen am ventralen oder dorsalen Sporn) zu vermeiden [5–7].

#### Welche Therapieoptionen stehen unter Berücksichtigung des Korrekturpotenzials und der unterschiedlichen Frakturtypen zur Verfügung? Welche wählen Sie im konkreten Fall?

- Konservative Behandlung:
  - Typ I: Gipsbehandlung oder äquivalente Ruhigstellung,
  - Typ II (bis zum 6. Lebensjahr) Eine Antekurvationsfehlstellung kann akzeptiert werden; hierbei kann z. B. eine Blount-Schlinge oder ein „Cuff-and-collar“-Verband als indirekte Repositionshilfe die Fehlstellung initial minimieren oder sogar ausgleichen.
- Operative Behandlung:
  - Typ-II-Frakturen mit Rekurvationsfehlstellungen sind instabil und bedürfen der operativen Versorgung.
  - Typ-III- und Typ-IV-Frakturen werden operativ stabilisiert, wobei die K‑Draht-Osteosynthese bevorzugt wird. Alternativ kann der Fixateur externe angewendet werden. In manchen Ländern (z. B. Frankreich) wird auch eine Versorgung mithilfe des „elastic stable intramedullary nailing“ (ESIN) bevorzugt (im deutschsprachigen Raum unüblich).
    Die Stabilität der gekreuzt eingebrachten Drähte ist höher als unilateral eingebrachte Drähte; drei Drähte (2 von radial und einer von ulnar) sind biomechanisch stabiler als 2 Drähte. Bei kleinen Kindern reichen 2 gekreuzte Drähte vollkommen aus [8, 9].

Bei operativem Vorgehen wird eine iatrogene Läsion des N. ulnaris in bis zu 10 % der Fälle angegeben [10]. Hier gibt es 2 unterschiedliche Auffassungen, wie damit umzugehen ist: Entweder werden rein sensible Läsionen belassen, aber bei zusätzlichen motorischen Läsionen wird die direkte operative Revision durchgeführt. Oder es wird auch beim kompletten Ausfall des Nerven zugewartet. Generell haben die Schäden des N. ulnaris eine gute Prognose. Insgesamt ist mit einer Rekonvaleszenzdauer von 3 bis 9 Monaten zu rechnen [6, 7, 10].

##### Der Fall.

Im vorliegenden Fall spielt das mögliche Korrekturpotenzial keine Rolle. Der Frakturtyp IV stellt eine klare Operationsindikation dar. Die Notfallindikation ergibt sich durch die erhebliche Dislokation und die Perfusionsstörung. Diese kann entweder durch eine direkte Gefäßläsion bedingt sein oder entstand indirekt durch Fragmentdruck auf die A. brachialis.

**Figure 5:**
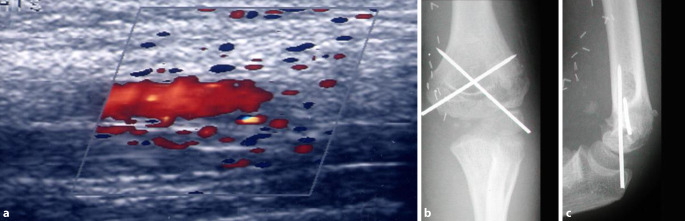

Im Anschluss an die Reposition und K‑Draht-Fixation war die Durchblutung jedoch immer noch aufgehoben, sodass eine intraoperative Duplexsonographie durchgeführt wurde, die einen persistierenden Perfusionsabbruch ergab (Abb. 5a). Es erfolgte das intraoperative Hinzuziehen der Gefäßchirurgen, die eine Revaskularisation mithilfe eines Gefäßinterponats durchführten (Abb. 5b).

#### Welche posttraumatischen Komplikationen können eintreten?

Die häufigste posttraumatische Deformität nach suprakondylärer Fraktur ist der Cubitus varus. Das funktionelle Ergebnis wird, wie in Tab. 1 dargestellt, klassifiziert.

- Eine relevante Varusfehlstellung findet sich in etwa 3 % der Fälle [11]. Valgusfehlstellungen basieren meist auf Läsionen im Bereich des Condylus radialis und führen hin und wieder auch zu Pseudarthrosen in diesem Bereich. Gelingen die Einstellung und Fixierung des Gelenkblockes am distalen Humerus unzureichend, resultiert meist eine mehrdimensionale Fehlstellung mit Varusdeformität, Innenrotationsfehler und Antekurvationsstellung. Eine Indikation zur Korrektur ergibt sich aus Funktionsstörungen, dem kosmetischen Erscheinungsbild und gelegentlichen Nervenirritationen.
- Zur Vorbereitung gehören zunächst die exakte Erfassung der Deformität mit Bestimmung der Abweichung sowie die Messung der Bewegung und Überprüfung der Gelenkfunktion. Deformitäten nach suprakondylärer Humerusfraktur führen bei Antekurvation zu einer Beuge- und bei Rekurvation zu einer Streckhemmung.
- Operative Korrekturmethoden:
  - Die operationstechnische Vorgehensweise zur Korrektur einer Valgus- bzw. Varusdeformität richtet sich nach dem Alter des Kindes. Es muss dabei zwischen Osteosynthesetechniken und Korrekturtechniken zur Osteotomie unterschieden werden. Bei Adoleszenten und nahe dem Wachstumsabschluss kann entsprechend der Korrektur beim Erwachsenen eine stabile Doppelplattenosteosynthese erfolgen. Für die jüngeren Kinder wird zur Stabilisierung – abhängig von der Korrekturtechnik – die Kirschner-Draht-Osteosynthese oder der Fixateur externe verwendet [12].
  - Ein häufiges Verfahren stellt die Behandlung im Fixateur externe mit dreidimensionaler suprakondylärer Derotationsosteotomie dar. Der Fixateur externe wird i. Allg. von Kindern gut toleriert.

**Table 1:** 

0 ≙ ideal	Keine Achsabweichung a.-p., seitlich gut	Kein Funktionsdefizit, Achsen seitengleich
1 ≙ gut	Bis 5° Varus/Valgus, Ante‑, Rekurvationsfehler bis 10°	Bis 10° Defizit Extension/Flexion, Achsfehlstellung bis 5°
2 ≙ tolerabel	5–10° Varus/Valgus	10–20° Defizit Extension/Flexion Valgus und Varus 5–10°
3 ≙ schlecht	10° Varus-Valgus oder mehrere Fehler	20° Differenz in einer Ebene oder mehr

##### Der Fall.

Im vorliegenden Fall kam es zu einer anatomischen Frakturkonsolidierung; das funktionelle Ergebnis konnte als ideal klassifiziert werden. Nach einer Ruhigstellung von 2 Wochen in dorsaler Oberarmschiene und Röntgenkontrolle nach dieser Zeit erfolgte der Mobilisationsbeginn spontan. Die Implantatentfernung konnte nach 6 Wochen vorgenommen werden, bei symmetrischer Ellenbogenachse, freier Funktion und guter Perfusion des Arms zu jedem Zeitpunkt konnte die Behandlung nach 8 Wochen abgeschlossen werden.
